# Mutual Effects and Uptake of Organic Contaminants and Nanoplastics by Lettuce in Co-Exposure

**DOI:** 10.1021/acsagscitech.3c00600

**Published:** 2024-03-26

**Authors:** Michael Taylor Bryant, Jianhong Ren, Virender K. Sharma, Xingmao Ma

**Affiliations:** † Department of Civil and Environmental Engineering, Texas A&M University, College Station, Texas 77843, United States; ‡ Department of Environmental Engineering, Texas A&M University-Kingsville, Kingsville, Texas 78363, United States; § Department of Environmental and Occupational Health, School of Public Health, Texas A&M University, College Station, Texas 77843, United States

**Keywords:** microplastics, nanoplastics, lettuce, polystyrene, xenobiotics

## Abstract

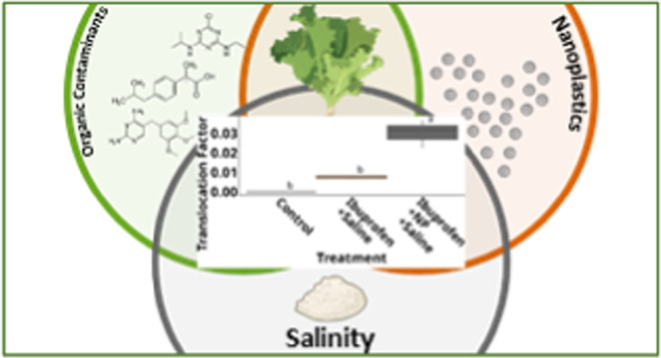

Organic contaminants, such as pesticides and pharmaceuticals, are commonly found in agricultural systems. With the growing use of plastic products, micro- and nanoplastics (MNPs) are increasingly detected in these agricultural systems, necessitating research into their interactions and joint effects to truly understand their impact. Unfortunately, while there has been a long history of research into the uptake of organic pollutants by plants, similar research with MNPs is only beginning, and studies on their mutual effects and plant uptake are extremely rare. In this study, we examined the effects of three agriculturally relevant organic pollutants with distinctive hydrophobicity as measured by log *K*_OW_ (trimethoprim: 0.91, atrazine: 2.61, and ibuprofen: 3.97) and 500 nm polystyrene nanoplastics on their uptake and accumulation by lettuce at two different salinity levels. Our results showed that nanoplastics increased the shoot concentration of ibuprofen by 77.4 and 309% in nonsaline and saline conditions, respectively. Alternatively, organic co-contaminants slightly lowered the PS NPs uptake in lettuce with a more pronounced decrease in saline water. These results underscore the impactful interactions of hydrophobic organic pollutants and increasing MNPs on a dynamic global environment.

## Introduction

Plastic is a defining legacy of the Anthropocene era.^1^ Over 350 million tons of plastics are currently produced worldwide,^2^ 90% of which is left unrecycled,^3^ causing an ever-growing thumbprint on the natural world. Very few biological processes can completely mineralize plastics, resulting in the growing pervasiveness of plastic wastes in the environment.^4−6^ Instead of disappearing, a new class of pollutant named micro- and nanoplastics (MP and NP, respectively, and micro- and nanoplastic (MNP) collectively) and defined as plastic particles with dimensions less than 5 mm and 1 μm,^7,8^ respectively, is generated through natural weathering such as photo-oxidation and mechanical abrasion.

Research on MNP in air and water has been performed extensively,^9−11^ highlighting their prevalence and risks to humans. Soil, however, may be the ultimate sink for plastic contamination, with up to 23 times higher plastic concentrations than even in the ocean.^12^ This generates a more nebulous risk of MNP exposure through accumulation in food crops and their consumption by humans, attracting substantial attention.^13,14^ One recent survey underscores the gravity of this risk, revealing extensive MNP contamination in supermarket produce, with average concentrations of roughly 10^4^ particles per gram of tissue attributed to processing and handling practices.^15^ Contamination after harvest is not the only potential source for MNP in produce. A growing list of reports has highlighted the accumulation of MNPs by several important agronomic and horticultural crops, including wheat,^16^ rice,^17^ strawberry,^18^ and cucumber^19^ through plant root uptake, further underlining the ubiquitous impact MNPs on global food production and food safety even though detailed uptake mechanisms are still unclear.

As pervasive as MNP contamination is in the environment, another category of pollution, such as contamination by organic pollutants, has a much longer history. In agricultural soils, pesticides, fertilizers, and pharmaceutical and personal care products are frequently detected.^20^ Plant uptake of these organic contaminants has long been established as primarily influenced by the compound’s hydrophobicity.^22^ Compounds with log *K*_OW_ greater than 1 but less than 3 diffuse well into the cell membrane and then into the cytosol, bypassing the major barrier between the cell and its environment. From there, they may rapidly move into the transpiration stream and then into the shoot tissue of the plant. For compounds with log *K*_OW_ greater than 3, they are typically retained in the cell membrane and gradually accumulate at the site of exposure while those with log *K*_OW_ less than 1 show poor affinity to plant roots and are not taken up by plants. From the perspective of food safety, these chemicals pose relatively low risks to humans via consumption of aboveground food crop tissues. However, this general trend of contaminant uptake might be altered by co-present MNPs because many previous studies have demonstrated that small particles such as engineered nanoparticles can markedly alter the uptake and transport of co-present organic contaminants.^21^ The question thus becomes how the established behavior of these organic contaminants in agriculture changes with the co-presence of emerging MNPs. This question is important for food safety because altered plant accumulation of organic contaminants may increase the risks of human exposure. Unfortunately, very few studies have examined the potential effect of coexposed MNPs on plant uptake of organic contaminants and how the interactions of these co-contaminants may differ with the properties of the organic compounds and more broadly with environmental conditions.

Global climate change has caused more frequent occurrence of widespread drought,^23,24^ making supplemental irrigation, often containing salt concentrations much higher than in rainfall,^25^ a necessity. This results in an increase in salinity in the soil over repeated irrigation cycles. High salt content will not only stress plants physiologically but also affect the interactions of coexisting environmental chemicals and MNPs, rendering it a critical consideration in the interactions of these contaminants. The objectives of this study included: (1) elucidating the mutual impact of three organic contaminants, with distinctive hydrophobicity, and MNPs on their uptake and accumulation in plants and (2) ascertaining the effect of salt stress on these interactions. Three commonly detected organic contaminants including ibuprofen, trimethoprim, and the herbicide atrazine, chosen for their range in hydrophobicity and thus variation in uptake behavior in plants, were used as model organic contaminants. 500 nm polystyrene (PS) NPs and lettuce were used as model MNPs and plants, respectively, due to size limitations on plant uptake of NP^13,26^ and because PS is one of the most frequently detected MNPs in the environment^2,27^ while lettuce is a popular plant species in the studies of plant accumulation of environmental contaminants.^15,28−31^

## Materials and Methods

### Chemicals and Plant Materials

PS NPs with a diameter of 500 nm were purchased from Sigma-Aldrich (NJ) as a 10% (m/m) dispersion. Particle size and size distribution were measured using ImageJ (version 1.53t24) after they were examined under a field emission scanning electron microscope (FE-SEM) (Figure S1, 505 ± 60 nm). Modified Hoagland salts were purchased from USBiologic (MA). Macerozyme R-10, a mixture of cellulase, hemicellulase, and lipase, was purchased from RPI (IL). 2-(*N*-Morpholino)ethanesulfonic acid (MES), a surfactant and pH buffer for the enzymatic digestion solution, trimethoprim, atrazine, ibuprofen, and sodium chloride were purchased from Sigma-Aldrich (MO). Log *K*_OW_ and other physiochemical properties of three concerned organic compounds are summarized in Table S1. High-performance liquid chromatography (HPLC) grade acetone and methanol as well as activated charcoal were purchased from Thermo Fisher (MA). Green leaf lettuce was purchased from a local supermarket. Fusion M1 lettuce seeds were purchased from Johnny’s Select Seeds (ME).

### Lettuce Growing Conditions

Lettuce seeds were sterilized for 10 min using a 2% bleach solution (Chlorox, CA) and then rinsed thrice using ultrapure water. Seeds were sown in batches of 20 on moistened filter paper in disposable Petri dishes. Germination of the seeds occurred over 4 days, with 2 days in the dark and 2 days under 16:8 day (30 °C):night (22 °C) cycle, until cotyledons had emerged and radicals were at least 2 cm long. After germination, seedlings were transplanted to 50 mL falcon tubes wrapped in foil and filled with 1/4 Modified Hoagland solution. Plants were grown for 21 days under the same light and temperature conditions as germination, and the hydroponic solution was refilled as necessary.

### Experimental Conditions

21-day-old lettuce seedlings were washed using excess ultrapure water, and then the seedlings were transferred to new 50 mL falcon tubes wrapped in foil containing different treatment solutions. Exposure treatments were performed using a complete cross-classification of three treatments: 10 mg/L PS NP dispersion (plastic); a combined mixture of trimethoprim, atrazine, and ibuprofen each at 1 mg/L in solution (contaminant mixture); and 100 mM NaCl (saline). Thus, there were 7 treatments: plastic, contaminant mixture, saline, plastic+contaminant mixture, contaminant mixture + saline, plastic + saline, and plastic+contaminant mixture + saline. In addition, an ultrapure water treatment was included as a control. Six seedlings were included in each treatment. The treatment solutions were topped up to 50 mL every 48 h, and the solution volume added to each tube was recorded at the time of watering. Hydroponic system was adopted in lieu of the soil growth system to avoid the compounding effect of soil in this early stage of investigation. Moreover, hydroponic systems are playing an increasingly important role in food production, especially in urban communities.^32^ Plants were harvested after 7 days of treatment exposure.

### Harvest Procedure

At harvest, plant roots were washed with any remaining hydroponic solution using ultrapure water. Plants were then separated into shoot and root tissues. Fresh weights of these tissues were recorded, three replicates from each treatment were transferred to labeled brown paper sacks, and the rest three replicates were cut into 1 cm wide strips and transferred to 25 × 100 mm^2^ glass test tubes. Whole samples in brown paper sacks were dried in an oven at 60 °C until complete dryness, about 24 h, then the dry sample weights were measured prior to analysis for trimethoprim, atrazine, and ibuprofen. Cut samples were transferred to a freezer at −20 °C for 24 h prior to the analysis for NP content in these tissues.

### Quantification of Trimethoprim, Atrazine, and Ibuprofen in Lettuce Tissues

Quantification of trimethoprim, atrazine, and ibuprofen was performed following the method from Carvalho with slight modifications.^33^ Briefly, dry plant tissues were ground using a mortar and pestle. 10 mL of a 95:5 MeOH:acetone solution was added to the ground samples in a 15 mL centrifuge tube. Sample tubes were sonicated at 40 MHz (Branson 5800, Branson Ultrasonics, CT) for 30 min and then centrifuged at 5000 g for 10 min. The supernatant was transferred to new centrifuge tubes with 0.25 g of activated charcoal, and then the same sonication and centrifugation steps were repeated. The supernatant was again collected and filtered through a 0.2 μm PTFE membrane. The samples dried at 60 °C under continuous flow of N_2_, then resuspended in 1 mL MeOH, and analyzed by HPLC (Dionex UltiMate 3000, Thermo Fisher Scientific, MA).

The HPLC method utilized two eluents, 100% HPLC-grade MeOH (A) and 0.5% o-phosphoric acid in DI water (B). Solution B was ramped from 5 to 80% over the first 0.5 min, then increased to 100% over the next 4 min, and held constant for 4 min. Solution B was then ramped back down to 5% over the remaining 4 min of the method. Recovery times and limits of detection for the three compounds using the method are found in Table S2.

### Quantification of Polystyrene Nanoplastics in Lettuce

PS NPs in plant tissues were quantified using enzymatic digestion, followed by FE-SEM imaging. The extraction method is based off our lab’s prior work extracting gold nanoparticles from tomato plants.^34^ Briefly, 2 g/L Macerozyme R-10 solution with MES buffer was added to the thawed tissue, then agitated at 300 rpm at 37 °C for 24 h. Afterward, the samples were filtered through a Whatman GF-D filter (pore size = 1.2 μm, Whatman, MA) to remove residual plant tissue. The filter was rinsed once with ultrapure water, and the mixture of the rinsate and filtrate was diluted to 100 mL. 5 mL of the final dilution was filtered through a 0.2 μm nitrocellulose membrane to retain extracted PS NPs. The membrane was washed with 10 mL of 50% MeOH and then fully dried in a desiccator before imaging by FE-SEM (JSM7500, RRID:SCR_022202). Detailed extraction procedures and materials are summarized in Text S1.

For the imaging, 1 cm^2^ of the dried membrane was cut from the center and then sputter-coated with 5 nm of Pt/Pd alloy. Imaging conditions were 5 kV acceleration voltage, 5 mA emission current, and a working distance of 8 mm. Ten images of the filter surface were taken and analyzed for the presence of PS NPs, the number of which were normalized to the viewing area, sample volume, and fresh weight tissue mass, resulting in a particle concentration (# of PS/g_FW_) as per eq 1. Final extraction values were compared to a standard curve developed using plastic control samples (Figure S2), prepared by injecting a known volume of stock PS NPs into lettuce tissue grown in 1/4 Hoagland solution.
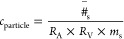
1where #®_s_ is the average particle count observed in the SEM viewing area (unitless); *R*_A_ is the ratio of viewing area to filter surface area (unitless); *R*_V_ is the ratio of subsample volume to total dilution volume (unitless); and *m*_s_ is the mass of fresh tissue used in the PS NP extraction (grams of fresh tissue).

## Results and Discussion

### Plant Biomass

**Figure 1:**
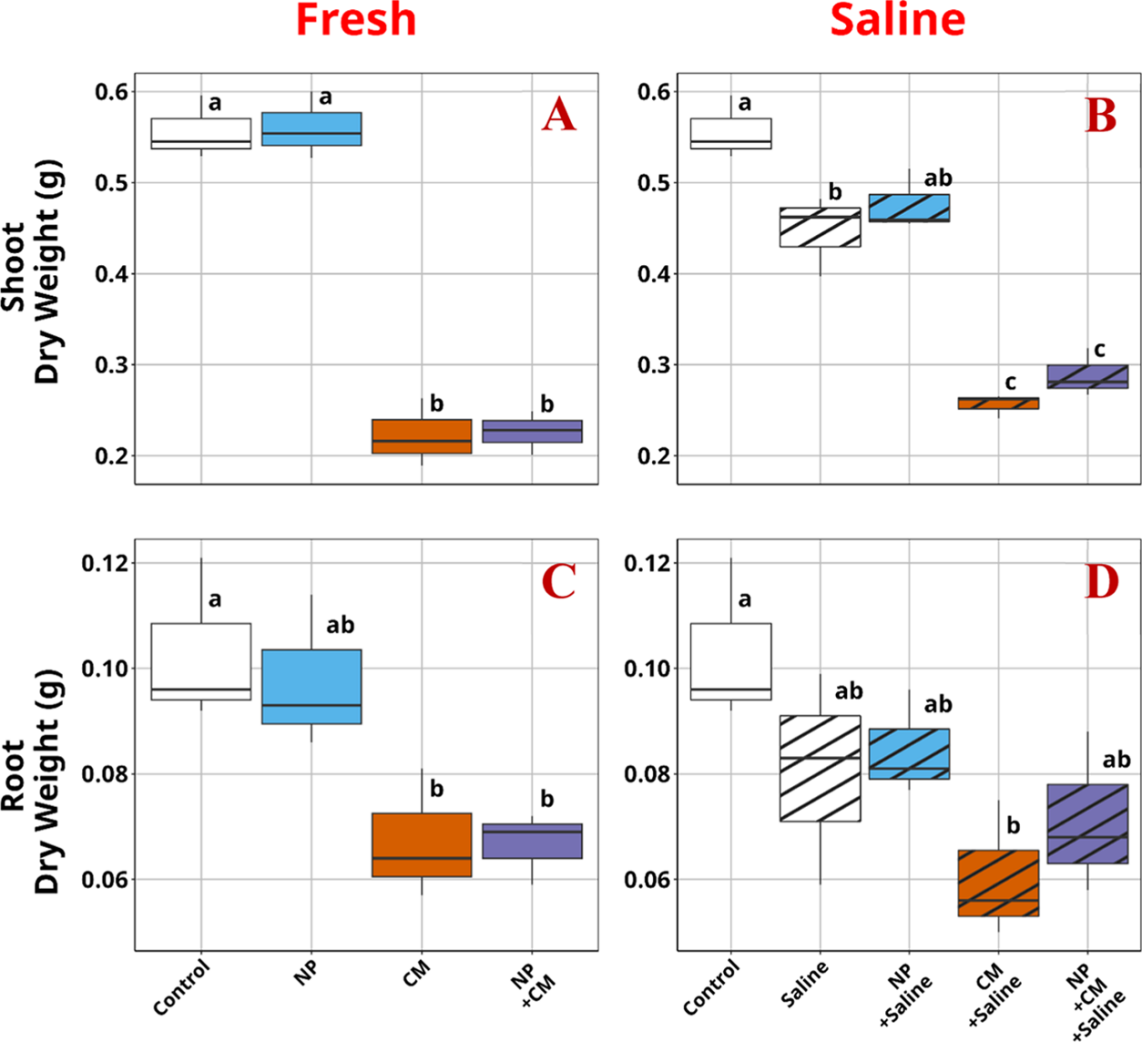
Dry weight of lettuce tissues. (A) Shoot tissue under freshwater exposure. (B) Shoot tissue under saline water exposure. (C) Root tissue under freshwater exposure. (D) Root tissue under saline water exposure.NP: plastic, CM: contaminant mixture.

Across treatment variables, plant health parameters tended to decrease with increasing numbers of stressors sourced from high salinity, PS NP, and contaminant mixture exposure effects. These effects are most notable for the tissue dry mass (Figure 1), with similar but largely nonsignificant effects seen on the tissue fresh mass (Figure S3). Without the effect of high salinity, the mixture of three organic contaminants significantly decreased the plant dry biomass by 35% for the root and 60% for the shoot. In contrast, PS NPs showed a minimal effect on the dry biomass alone or synergistically with the contaminant mixtures.

Compared with the nonsaline treatments, high salinity and contaminant exposure are much more impactful. The shoot and root dry masses were reduced by 20 and 22%, respectively, by the salt stress alone, as compared to the control under nonsaline conditions. The co-occurrence of salt stress and the three contaminant mixtures further significantly lowered the dry root biomass by 41% and shoot biomass by 54% compared with plants growing in high saline water alone, suggesting some synergistic effect. Interestingly, PS NPs did not demonstrate any negative effects on plant biomass in the presence of these stresses. In fact, the presence of PS NPs slightly improved the plant dry biomass when plants are stressed by high saline water and organic contaminants.

### Plant Uptake of Organic Contaminants

Trimethoprim, with a log *K*_ow_ of 0.91, was below the detection limit in both the root and shoot tissues of any treatments, regardless of the presence or absence of PS NPs. This result is consistent with the general observation of the low accumulation of very hydrophilic compounds in plant tissues and is supported by other research showing very low uptake of trimethoprim in lettuce grown in sandy media,^28^ with their values very near to our method’s limit of quantification (Table S2).

**Figure 2:**
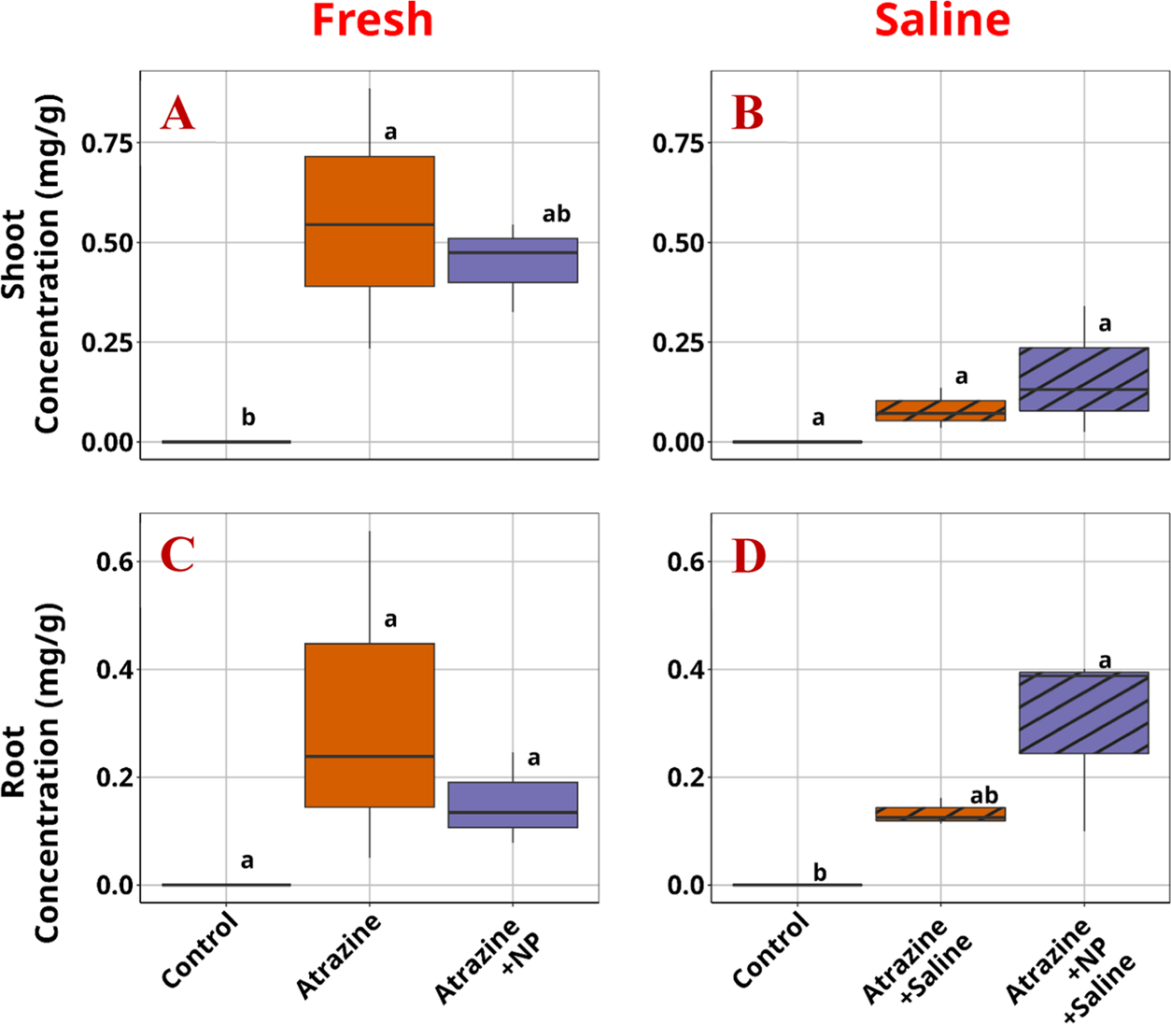
Atrazine concentrations in lettuce tissues. (A) Shoot tissue under freshwater exposure. (B) Shoot tissue under saline water exposure. (C) Root tissue under freshwater exposure. (D) Root tissue under saline water exposure.NP: plastic.

Atrazine uptake by lettuce root and translocation into shoot tissue was largely unaffected by PS NPs, although saline exposure alone decreased the concentration of atrazine in the shoot tissues by approximately 85% (Figure 2). Interestingly, while PS NPs did not significantly alter plant atrazine uptake, some differences in their interaction with atrazine were noticed in the fresh and saline water. In freshwater, PS NPs slightly lowered the concentration of atrazine in both lettuce roots and shoots; however, in saline water, PS NPs increased the concentration of atrazine in roots by 122% and in shoots by 105% compared with plants grown in saline water with the presence of atrazine alone.

**Figure 3:**
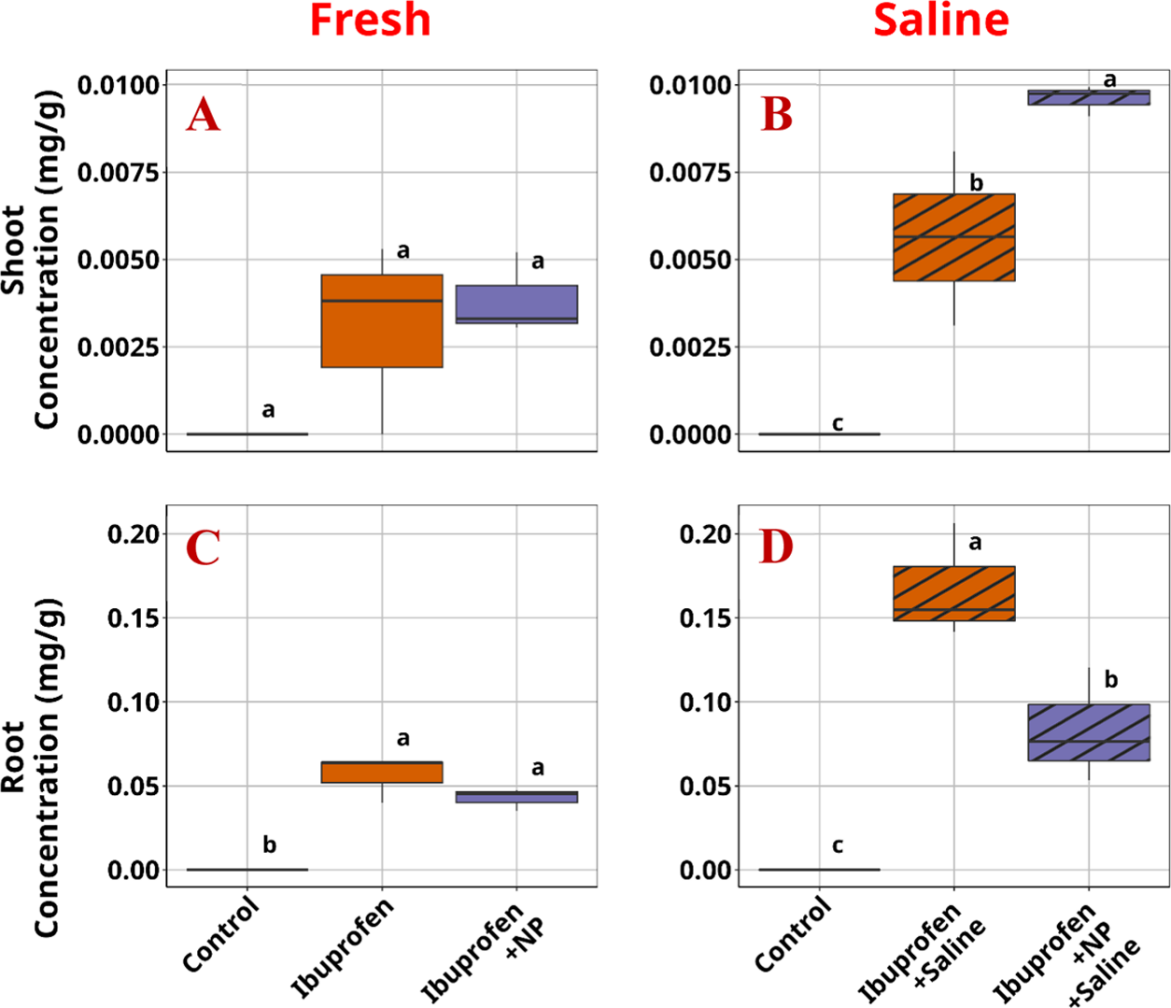
Ibuprofen concentrations in lettuce tissues (A) Shoot tissue under freshwater exposure. (B) Shoot tissue under saline water exposure. (C) Root tissue under freshwater exposure. (D) Root tissue under saline water exposure. NP: plastic.

**Figure 4:**
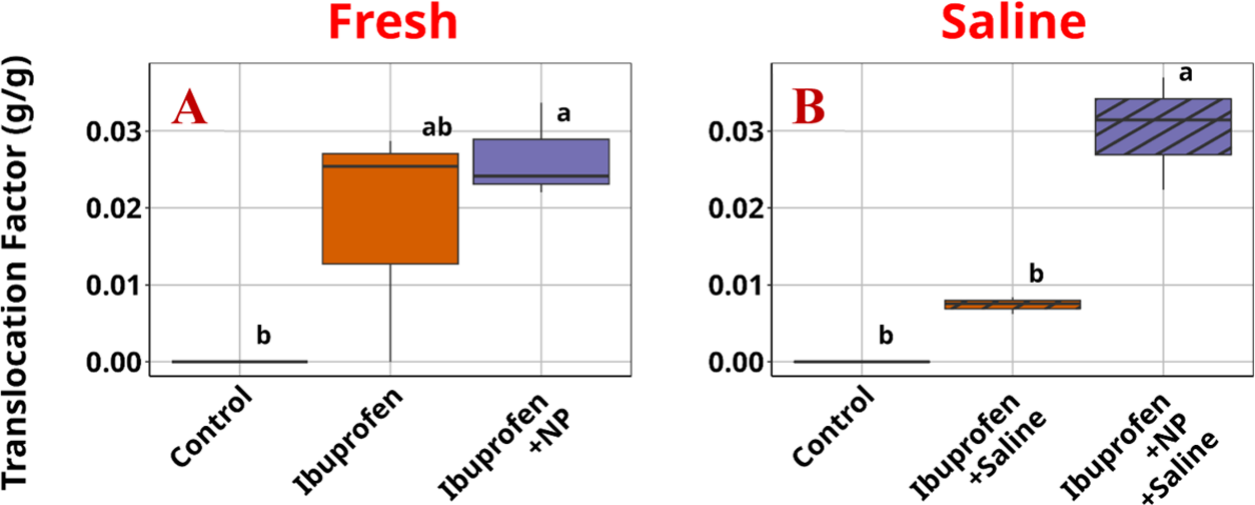
Translocation factor of ibuprofen in the presence of NPs in (A) fresh and (B) saline water. NP: plastic.

In contrast to the behavior shown by atrazine, ibuprofen, with a higher log *K*_ow_, showed a much lower shoot concentration, in agreement with the expected poor in planta transport of highly hydrophobic compounds (Figure 3). Upon exposure to the salinity treatments, the root concentration of ibuprofen increased by almost three times, likely due to the effect of ionic strength lowering the solubility of ibuprofen, already near saturation in the experiment (∼21 mg/L at 25 °C). Combining this exposure with PS NP, a significant increase of ibuprofen concentrations in the shoot and a concomitant decrease in the root concentration was observed in the saline water This effect was not observed in the nonsaline treatments even though a slightly lower concentration of ibuprofen in lettuce roots was also noticed in the nonsaline media. The result suggests that PS NPs significantly increased the in planta root-to-shoot translocation factor of ibuprofen, defined as the ratio of ibuprofen in lettuce shoot versus its concentration in lettuce root, upon co-exposure with PS NPs and salinity stress, compared with the treatment with similar exposure to salinity and ibuprofen but without PS NPs (Figure 4). This translocation factor is even slightly higher than those observed in the nonsaline treatments, regardless of the presence or absence of PS NPs.

The observation of elevated uptake and translocation of highly hydrophobic compounds by PS NPs in saline water is of incredible importance because the very hydrophobic compounds found in agricultural soils would pose greater health risks due to the presence of MNPs that would otherwise be limited to the soil and root fractions. Our results suggest that increasing concentrations of MNPs and heightened salinity in agricultural soils may unexpectedly increase the concentration of hydrophobic compounds in food crops, endangering the health of the public. In addition to the accumulation of organic contaminants, the potential bioaccumulation of MNPs is also of concern. Unfortunately, the detection and quantification of MNPs in plant tissues is still in the fledgling stage, and no standard method is available in the literature. Therefore, we first developed a semiquantitative method for PS NPs detection in lettuce tissues and then evaluated the potential effect of xenobiotics on plant PS NPs uptake.

### Nanoplastic Quantification in Lettuce Tissues

**Figure 5:**
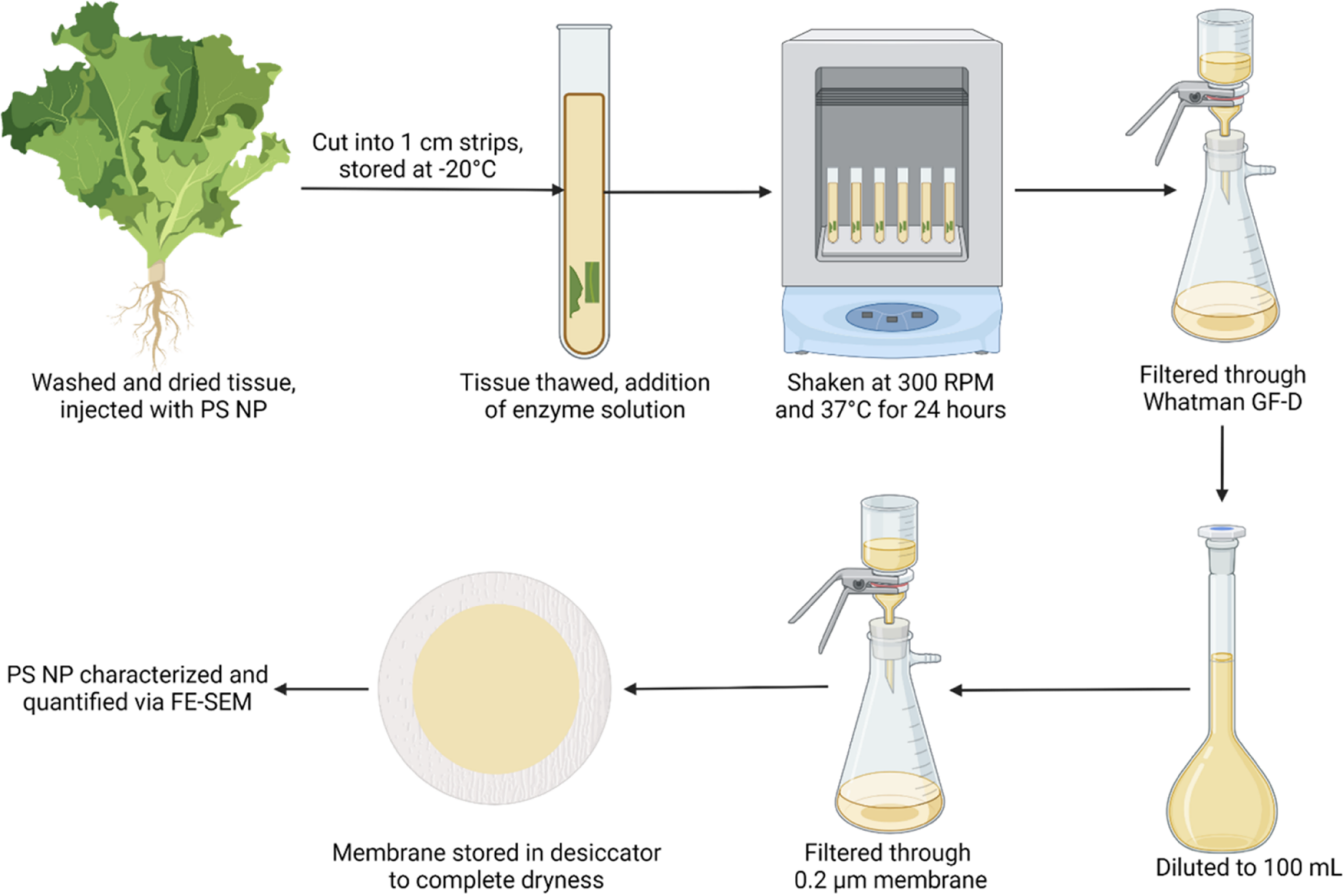
Schematic representation of PS NP extraction from lettuce tissue.

Using spiked lettuce tissues containing the equivalent of 2, 1, 0.5, and 0.1 mg/g PS NP per gram of fresh weight, a standard curve was created (Figure S2), and the limits of detection and quantification for this method were determined to be 4.35 × 10^–8^ and 1.15 × 10^–9^ particles/g of fresh tissue, respectively. Based on the particle size (505 nm), spherical shape, and density of PS, the mass concentration corresponding to the limit of detection and quantification of this method was estimated at 0.11 and 0.35 mg/g fresh tissue (eq S1), respectively. SEM imaging of PS NP controls revealed 17 cleanly defined particles for the lowest volume of PS NP stock injected into plant tissues, equivalent to 1.9 × 10^9^ particles per gram of plant tissue. Compared with the PS NP standard, the extraction efficiency of the method is 31.8 ± 15.7%. A detailed extraction and semiquantification of PS NPs in lettuce of the optimized method is summarized in Figure 5 and laid out in Text S1. To confirm the feasibility of the method, lettuce seedlings were grown in hydroponic solutions exposed to 10 mg/L PS NPs alone, and the results showed that PS NPs were mostly retained within the root tissue with some translocation to the shoot tissue. After counting sample images (Figure S4), the concentration of PS NPs in lettuce tissues was approximately 3.3 mg/g (4.8 × 10^10^ particles/g fresh root) and 0.1 mg/g (1.9 × 10^9^ particles/g fresh shoot). The results substantiated the feasibility of our method for MNP analysis in plant tissues and confirmed that NPs of up to 500 nm can be taken up by plant roots and translocated to shoots. Li et al.^19^ exposed cucumber plants to 50 mg/L of PS NPs (100 nm) hydroponically for 7 days and determined the concentration of PS NPs in plant tissues using pyrolysis gas chromatography–mass spectrometry (GC–MS). The reported concentrations of plastics in the cucumber plant tissues were 2.82 mg/g in root tissues and 0.29 mg/g in stem tissues (undetectable concentrations in the leaf). Our detected concentrations of NPs were very similar in root tissue (2.21 mg/g). We did not distinguish between stem and leaf tissue, however. Our shoot tissue concentration of 0.03 mg/g, while an order of magnitude lower than Li’s reported stem concentration, is still reasonable considering the tissue discrepancy and our use of PS NP that were 5 times larger in diameter. The relatively low extraction may be partly attributed to the aggregation of NPs in the filtration process, causing unintended retention of NPs by the filter. Nevertheless, our tissue concentrations showed similar results to those seen in cucumber after 7-day exposure,^19^ which was analyzed with a more expensive and complicated PY-GC/MS method. This confirms the feasibility of our method as a semiquantitative approach for NP detection in plant tissues. An advantage of our method is that it allows for the characterization of MNPs and their localization in the plant tissues of environmental samples when the method is combined with other spectroscopic methods. Using this method, we quantified the PS NPs in lettuce tissues from different treatments in this study.

### Plant Uptake of PS NPs

**Figure 6:**
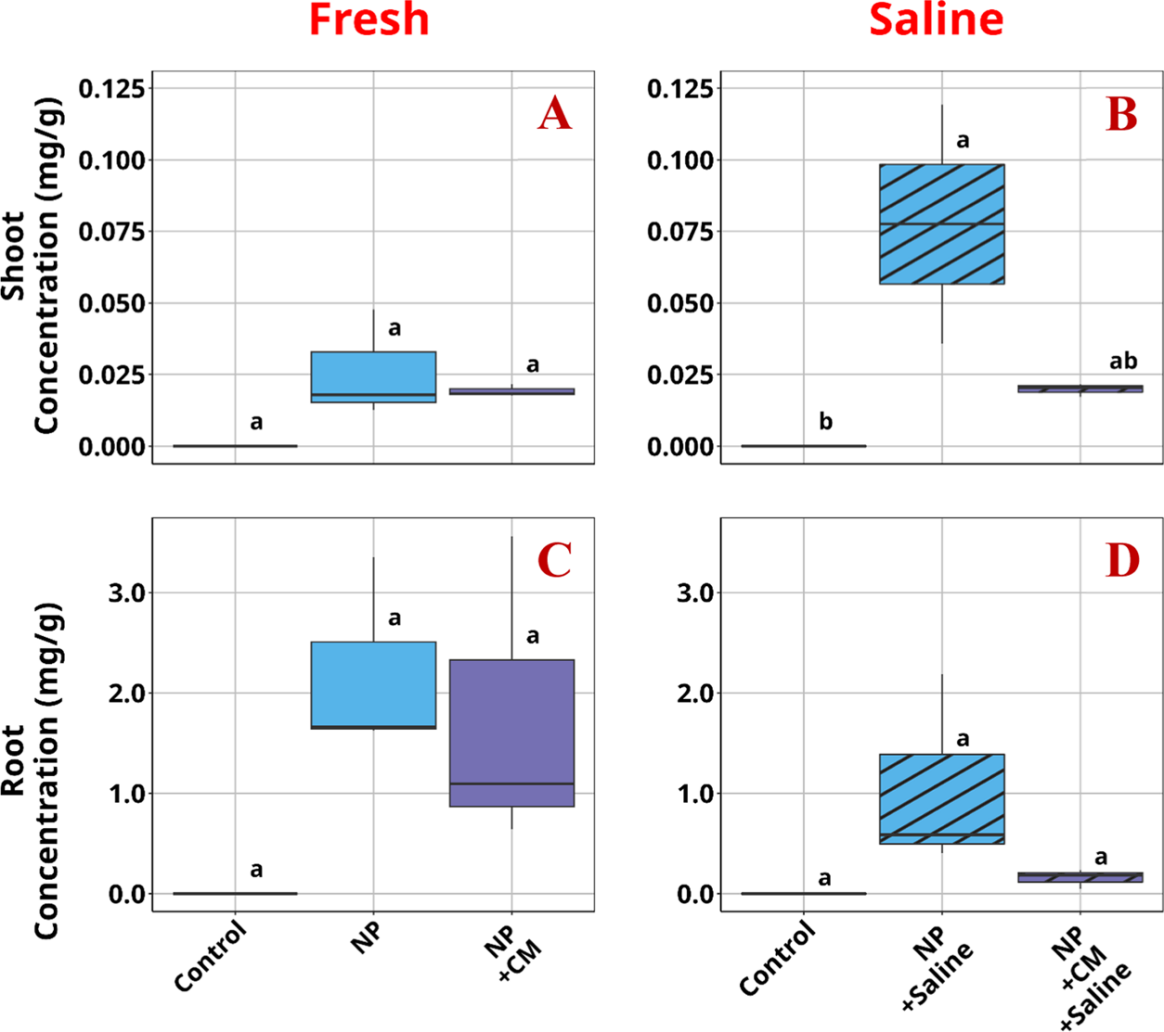
Nanoplastic concentrations in plant tissues. (A) Shoot tissue under freshwater exposure. (B) Shoot tissue under saline water exposure. (C) Root tissue under freshwater exposure. (D) Root tissue under saline water exposure. NP: plastic, CM: contaminant mixture.

**Figure 7:**
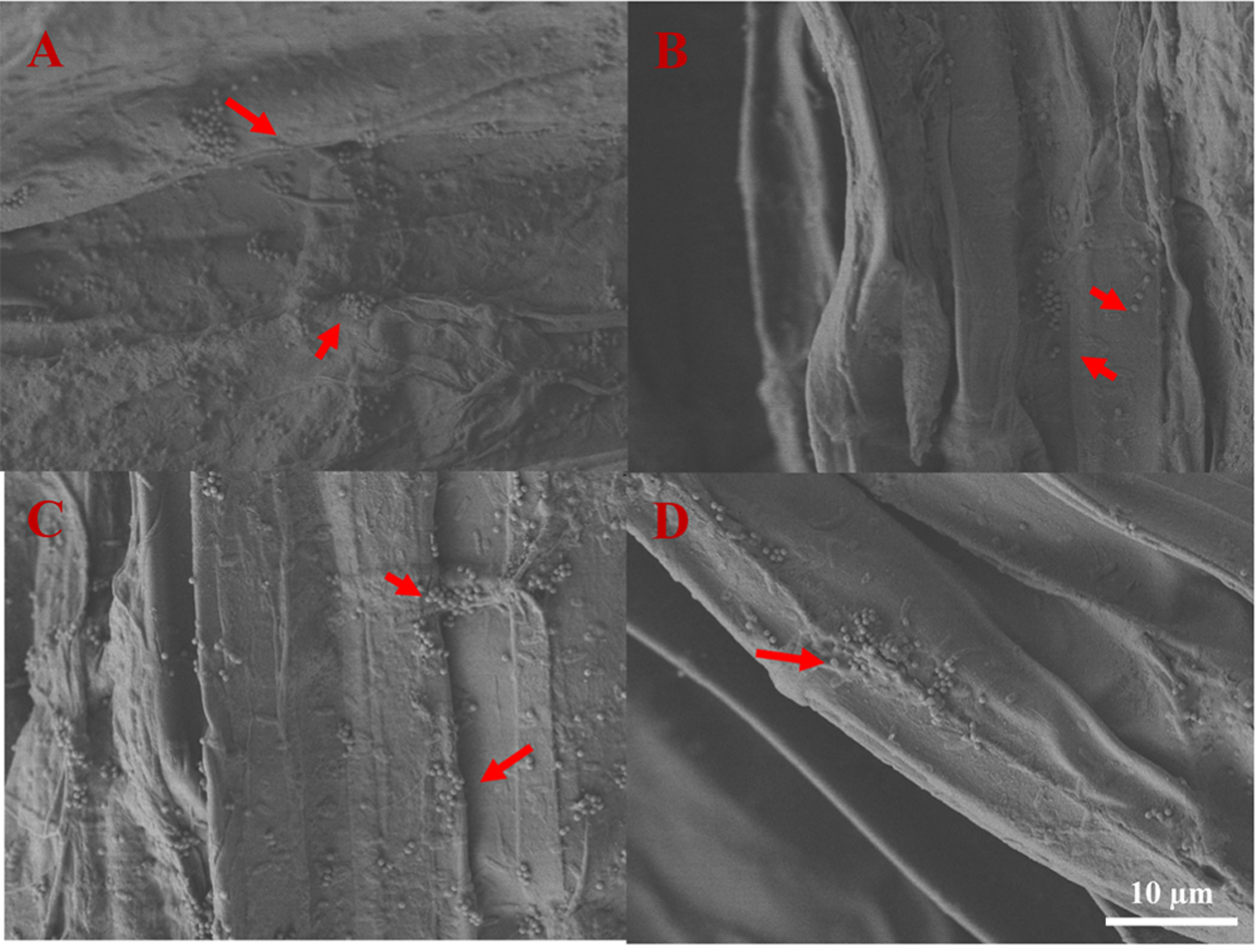
SEM images of lettuce root surface after 7-day PS NP exposure. (A) Unwashed root tissue. (B) After washing with ultrapure water. (C) After sonication for 10 min. (D) After sonication for 30 min. Red arrows indicate PS NPs.

PS NP uptake was observed in plant tissues that were exposed to the NPs. Statistical analysis of this uptake, however, showed that root uptake is not significantly different from the control even though the concentrations of PS NP were clearly elevated (Figure 6). Overall, combined exposure of PS NPs with salinity stress results in reduced uptake of NPs, likely due to lowered water uptake by the plant during the treatment exposure period (Figure S5). This agrees with previous research, showing NP uptake is a passive process tied to the tanspiration rate of the plant.^18^ Intriguingly, the presence of xenobiotics slightly lowered the concentration of PS NPs in lettuce roots in freshwater, and this impact was more noticeable in saline water. One possible reason is the synergistic effect of high salinity and xenobiotics further stressed the plants and lowered their plant biomass and water uptake (Figures 1, S5 and S6). Another possible reason could be the greater aggregation of PS NPs in saline water due to higher ionic strength, which can be further intensified by the presence of organic contaminants that dissociate at the solution pH (Table S1). This, however, seems less likely based on the SEM images of roots (Figure 7) processed differently after sampling at harvest. Based on the results, PS NPs attached to the root surface were primarily individual particles, and minimal aggregation was observed. These images also showed that PS NPs could not be fully removed by washing or even sonication; therefore, the root uptake of PS NP reported in this study is essentially a combination of truly taken up PS NPs and those merely attached to the surface of roots. To distinguish these two groups of NPs associated with plant roots remains a point of further research to help determine whether the observed changes in organic contaminant uptake are due to transport with the PS NP or if some other mechanism is involved. In summary, our study revealed the close interactions of growing MNPs with three organic co-contaminants and showed that PS NPs could markedly alter the plant uptake of hydrophobic compounds. This is likely the first study to explicitly investigate the mutual effects of NPs and organic co-contaminants on their plant uptake. Due to the diversity of plastic compositions, future studies should be expanded to other types of NPs to understand the possible compositional effect of plastics. Future studies should also be expanded to soil media, which, due to the adsorption of MNPs and organic contaminants on soil particles and potential interactions with local microorganisms,^35,36^ could display different mutual effects of MNPs and other contaminants. With the continued impact of global climate change and the increasing use of plastic products, a continuing examination into the synergistic interactions of contaminants and MNPs in an agricultural setting is imperative for sustainable agriculture and food safety.

## Supporting Information Available

Size analysis of PS NP used in this study; standard curve used in PS NP tissue concentration determination; fresh weight of lettuce root and shoot from different treatments; context of the enzymatic extraction method; solution uptake during exposure period; calculation to convert particle concentration to mass concentration of PS NP; selected physicochemical properties of organic contaminants used in this study; and HPLC limits of detection and quantification for organic contaminants used in this study (PDF)
