# Supplementary material for: Mutual Effects and Uptake of Organic Contaminants and Nanoplastics by Lettuce in Co-Exposure
Source: ACS Agric Sci Technol. 2024 Mar 26;4(4):463–70. doi: 10.1021/acsagscitech.3c00600 (PMC11022170; doi:10.1021/acsagscitech.3c00600)
Supplement: Supplementary file 1 — as3c00600_si_001.pdf [file as3c00600_si_001.pdf]

Mutual Effects and Uptake of Organic Contaminants and Nanoplastics by Lettuce in Co-exposure

Michael Taylor Bryant[1], Jianhong Ren[2], Virender K. Sharma[3], Xingmao Ma[1,*]

[1]Department of Civil and Environmental Engineering, Texas A&M University, College Station, TX, 77843

[2]Department of Environmental Engineering, Texas A&M University-Kingsville, Kingsville, TX, 78363

[3]Department of Environmental and Occupational Health, School of Public Health, Texas A&M University, College Station, TX 77843

***Corresponding Author:**

Dr. Xingmao Ma

Email: xma@civil.tamu.edu

Supplementary Figures: 6

Supplementary Equations: 1

Supplementary Tables: 2

Supplementary Texts: 1

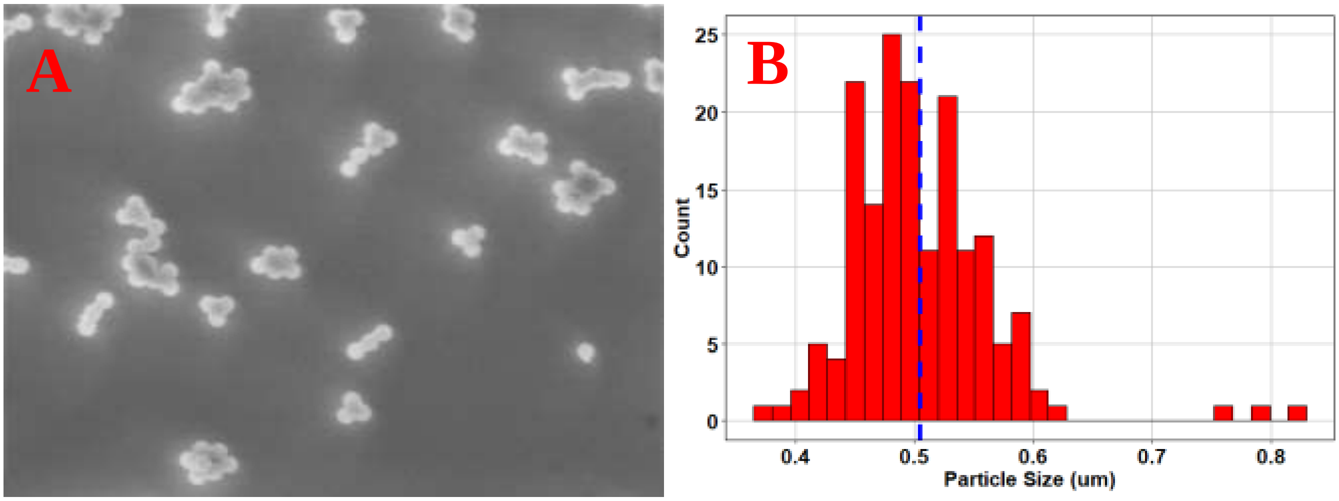

Figure S1. Commercially obtained 500 nm PS NP on p-Type Silicon Chip (A) and particle size distribution with mean 505 nm marked in blue (B).

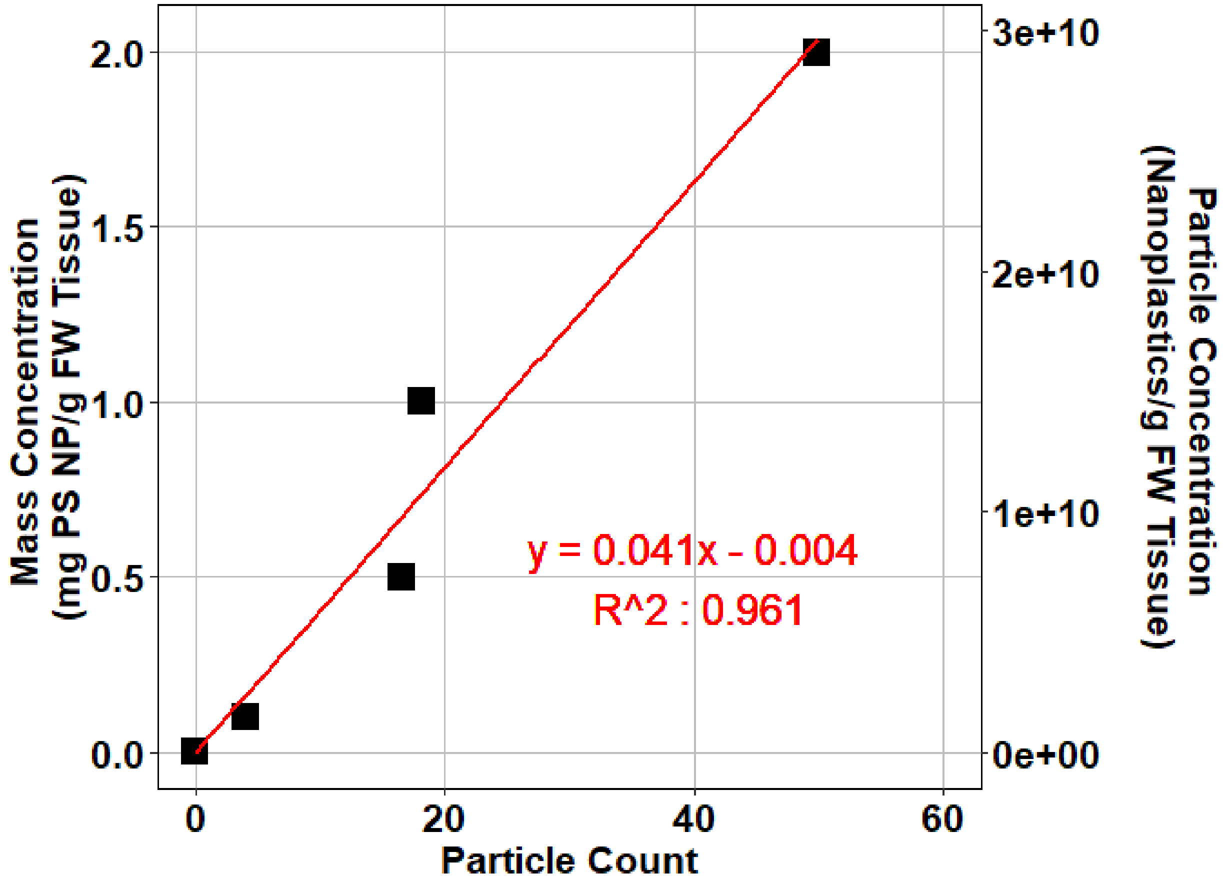

Figure S2. Standard curve for polystyrene nanoplastics in lettuce tissue. Y-axis values are concentrations injected into plant tissue (left) and extrapolations based on **Equation 1** (right). X-axis represents the PS NPs observed under the FE SEM. Displayed equation for calculation of mass concentration, y-intercept not significant ($p>0.05$).

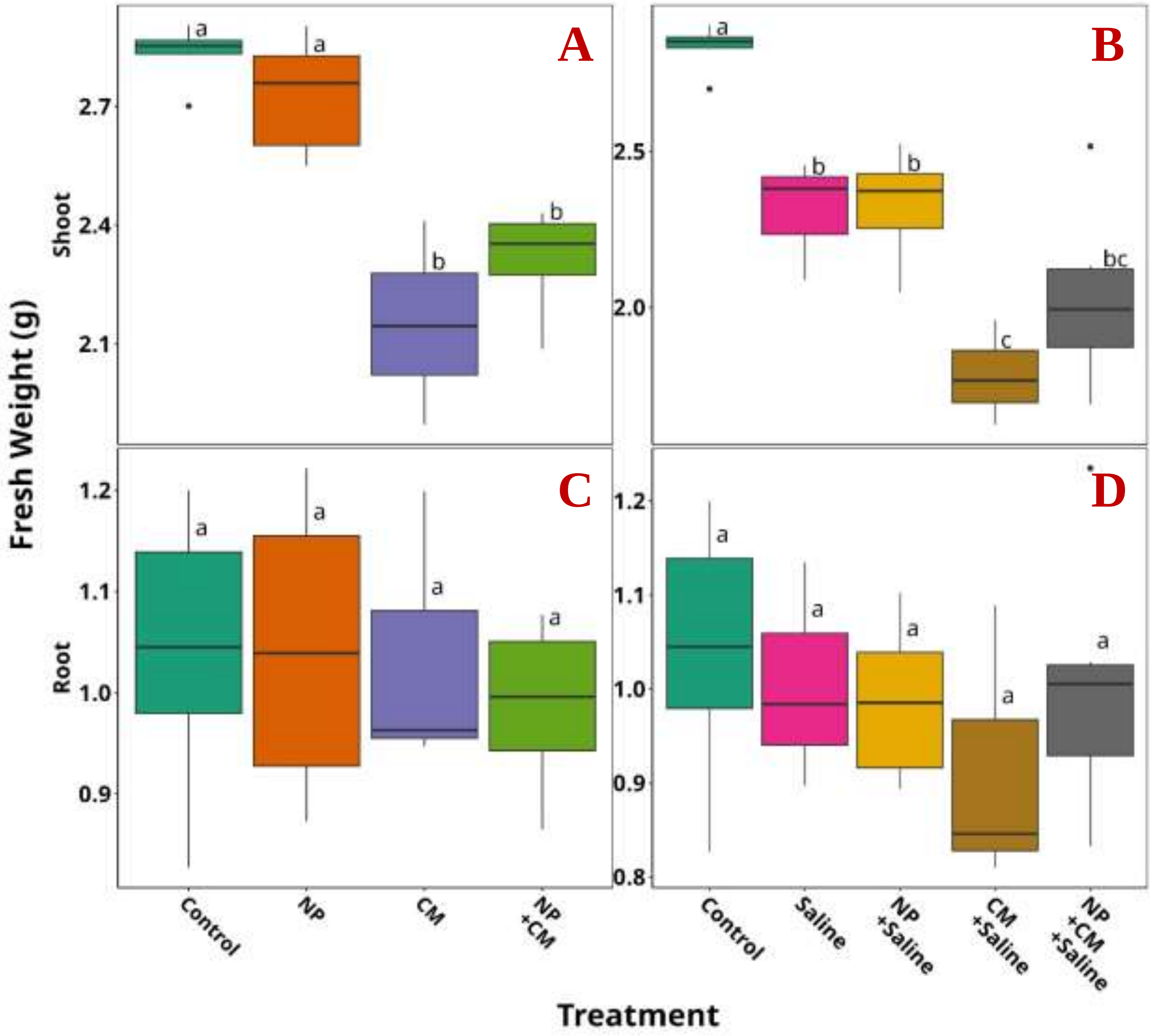

Figure S3. Fresh weight of lettuce shoot and root tissues under non-saline (A and C) and saline (B and D) conditions exposed to different combinations of PS NPs and xenobiotics. n=3. NP: nanoplastics, CM: contaminant mixtures.

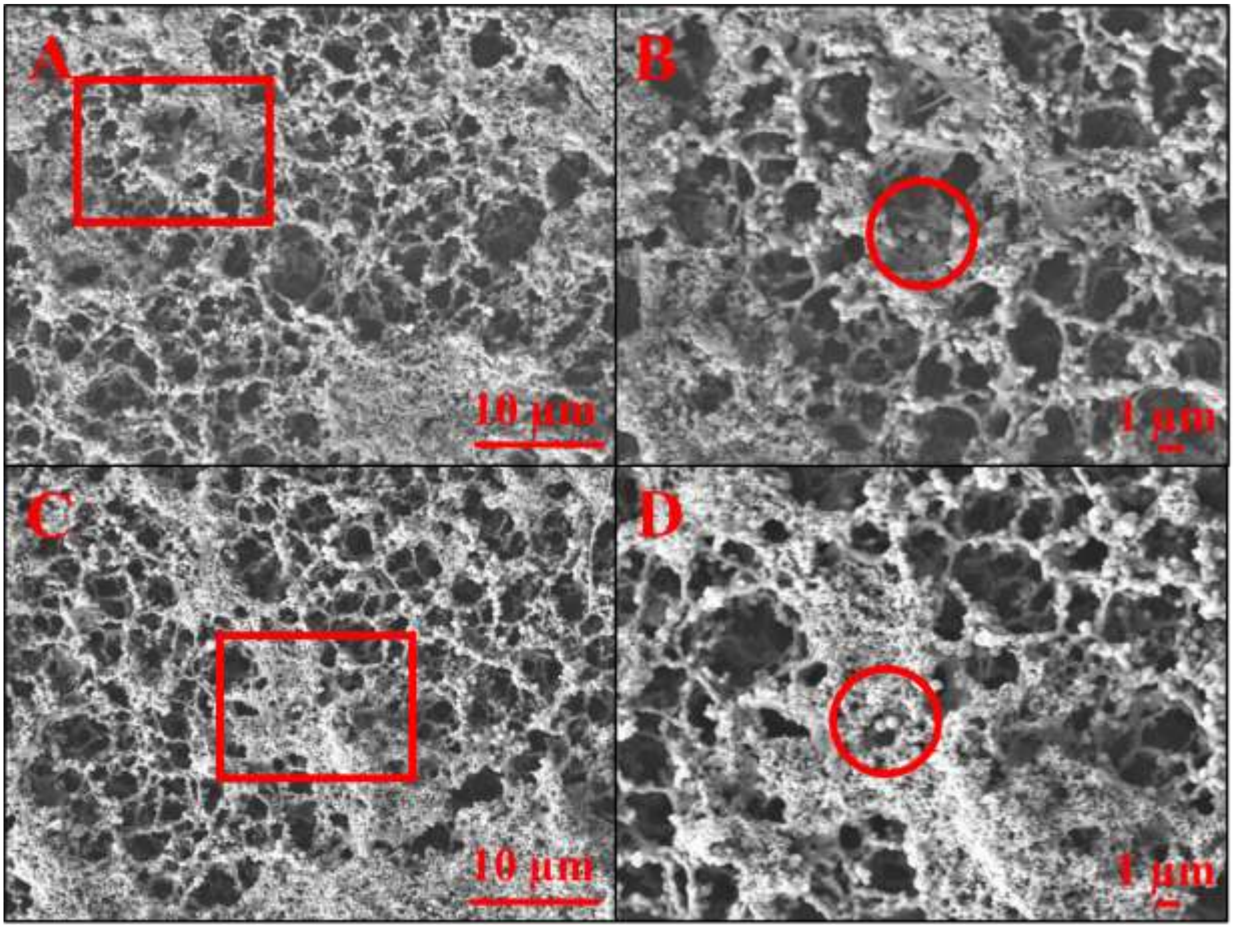

Figure S4. Representative SEM images of lettuce shoot (A and B) and root (C and D) filtrate after enzymatic extraction. B and D are 5000x magnification views of the regions indicated by the red box in A and C, respectively.

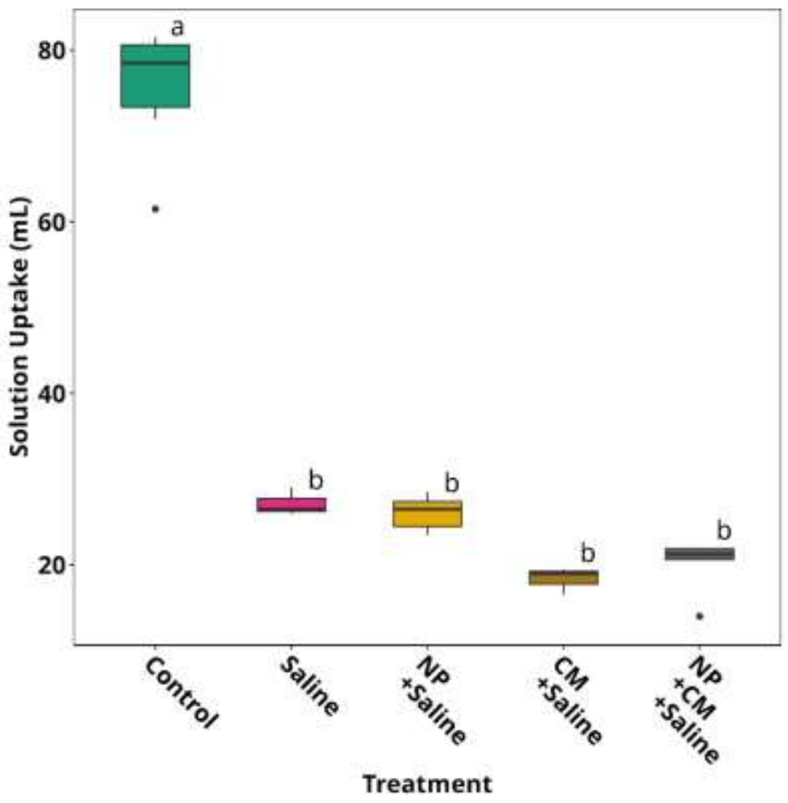

Figure S5. Water uptake by lettuce from different treatments. High water salinity significantly lowered the water uptake by lettuce. NP: nanoplastics, CM: contaminant mixtures. Different letters indicate significant differences, n=6.

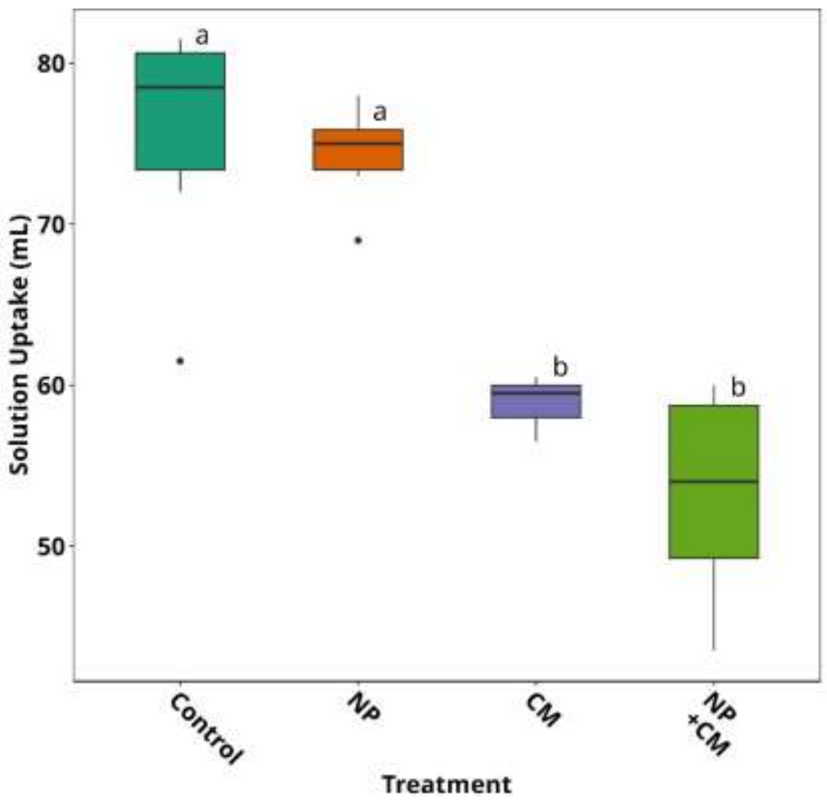

Figure S6. Water uptake by lettuce from different treatments. Organic contaminant mixture significantly lowered the water uptake by lettuce.

$$c_{PS} = c_{Particle} \times \frac{4}{3}\pi\bar{r}_{PS\ NP}^{3} \times \rho_{PS}$$ **Equation S1**

c_{PS}: Mass concentration of PS in plant tissue (mg PS per gram fresh tissue)

$c_{Particle}$: Particle concentration from Equation 1 (particle number per gram fresh tissue)

$\bar{r}_{PS\ NP}$: Average diameter of PS NP in SEM images (adjusted to cm)

ρ_{PS}: Density of PS (1.05 g/cm^3)

Table S1. Physicochemical properties of organic compounds used in study. Data from PubChem[1]

Compound	$LogK_{OW}$	pK_A	Molecular Weight (g/mol)	Structure
Trimethoprim	0.91	7.12	290.32	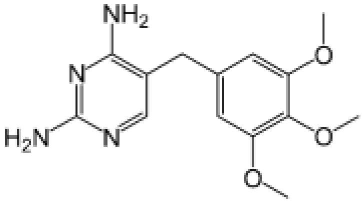
Atrazine	2.61	1.56	215.68	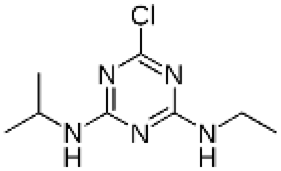
Ibuprofen	3.97	5.3	206.29	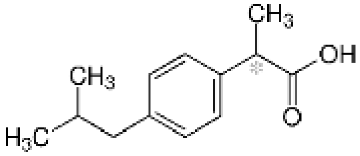

*Chiral center of Ibuprofen

Table S2. HPLC recovery times and limits of detection (LoD) and quantification (LoQ) for xenobiotics of interest. LoD and LoQ are based on the standard deviation of the lowest concentrations standard (sd) and the slope of the calibration curve (m) calculated as $\frac{3.3 \times sd}{m}$ and $\frac{10 \times sd}{m}$, respectively.

**Compound**	**Recovery Time (min)**	**LoD (µg/g)**	**LoQ (µg/g)**
Trimethoprim	3.7	2.8	8.6
Atrazine	5.3	3.3	10.1
Ibuprofen	6.5	0.4	1.2

Text S1 - Enzymatic Digestion of Plant Tissues and Preparation for Analysis by FE-SEM

Chemicals

2 g/L Macerozyme R-10 in 20 mM MES (adjusted to pH 5 with 0.4 M NaOH)

25% Methanol in RO water

RO water

Equipment

Fume hood

Electric scale

-20°C Freezer

Environmentally Controlled Incubator Shaker

Vacuum Filtration apparatus

47 mm Whatman GF-D filter

47 mm 0.2 µm Nitrocellulose membrane

100 mL volumetric flask

Disposable petri dish

Desiccator

Materials

Aluminum foil

Paper towels

16x100 mm glass test tubes

Scissors

Protocol (All steps performed under a fume hood unless samples are covered with foil)

1. With a sharp pair of scissors, separate root and shoot tissue of rinsed and dried lettuce samples. Rinse scissors between samples with RO water and dry with paper towel.
2. Record fresh weight of tissue samples using an electric scale. (Limit sample size to 2g)
3. With a sharp pair of scissors, trim tissue samples in 1 cm wide strips. Transfer to glass test tube and cover with foil.
4. Transfer samples to -20°C freezer and freeze for at least 24 hours.
5. Allow samples to thaw at room temperature. Remove foil, then for each gram of fresh weight tissue, add 20 mL of enzyme solution.

6. Transfer samples to an incubator shaker set to 37°C and 300 RPM. Leave for 24 hours.
7. At the end of 24 hours, sample by sample:
    a. Filter through Whatman GF-D filter using vacuum filtration apparatus. Rinse glass surfaces exposed to sample twice with RO water. Collect rinsate.
    b. Combine rinsate with filtrate and dilute to 100 mL with volumetric flask.
    c. With a clean vacuum filtration apparatus, filter 5 mL of the diluted sample through the 0.2 µm nitrocellulose membrane. Wash with 10 mL of the 25% methanol solution.
    d. Transfer nitrocellulose membrane to disposable petri dish, then dry in sealed desiccator for at least 24 hours.

Samples are ready for analysis by FE-SEM following protocol laid out by appropriate lab protocol. Use acceleration voltage 5 kV, emission current 5 µA, and working distance 8 mm for best images.

References

1. Kim S, Chen J, Cheng T, et al. PubChem 2023 update. *Nucleic Acids Res*. 2023;51(D1):D1373–D1380. doi:10.1093/nar/gkac956
